# Combined Treatment with Doxorubicin and Rapamycin Is Effective against In Vitro and In Vivo Models of Human Glioblastoma

**DOI:** 10.3390/jcm8030331

**Published:** 2019-03-09

**Authors:** Anna Lisa Iorio, Martina Da Ros, Claudio Pisano, Maurizio de Martino, Lorenzo Genitori, Iacopo Sardi

**Affiliations:** 1 Neuro-oncology Unit, Department of Pediatric Oncology, Meyer Children’s Hospital, 50139 Florence, Italy; martina.daros@meyer.it (M.D.R.); maurizio.demartino@unifi.it (M.d.M); lorenzo.genitori@meyer.it (L.G.); iacopo.sardi@meyer.it (I.S.); 2 BIOGEM Research Institute, 83031 Ariano Irpino, Italy; claudio.pisano@biogem.it

**Keywords:** doxorubicin, rapamycin, glioblastoma, mTOR inhibitor, chemotherapy

## Abstract

Despite numerous clinical trials, glioblastoma (GBM) remains a tumor that is difficult to treat. The aim of this study was to investigate the potential of a new pharmacological approach, combining doxorubicin (Dox) and rapamycin (Rapa), in in vitro and in vivo GBM models. Cytotoxic and anti-proliferative effects of Rapa *plus* Dox treatments were analyzed in GBM cell lines. The in vivo effectiveness of these treatments was investigated in an orthotopic xenograft mice model of GBM. In vitro results demonstrated that prolonged exposure to Rapa sensitize GBM cells to Dox treatments. In vivo results demonstrated that Rapa (5 mg/kg) *plus* Dox (5 mg/kg) determined the major tumor growth inhibition (−97.29% vs. control) but results in greater toxicity. The combination Rapa *plus* Dox (2.5 mg/kg) showed a tumor inhibition like Rapa *plus* Dox (5 mg/kg) with a toxicity comparable to Rapa alone. Thus, this study demonstrated the efficacy of this pharmacological approach, providing the rationale for a clinical application of this combinational therapy in “poor-responder” GBM patients.

## 1. Introduction

Glioblastoma (GBM) is the most frequent and lethal brain tumor in adults. Location, aggressiveness, and diffuse infiltrative growth make GBM therapy extremely challenging and frequently unsuccessful. Consolidated first-line treatment of newly diagnosed GBM consists of maximal surgical resection, when possible, followed by radiotherapy (RT) and adjuvant temozolomide (TMZ) [1]. This therapy provides a median overall survival of 12 months, and a five-year survival rate of less than 5% [2,3]. Such a poor prognosis generates a compelling need for innovative and effective therapeutic strategies.

Doxorubicin (Dox) is an anthracycline antibiotic with antineoplastic activity. This historical drug is often used, together with other chemotherapy agents, in many tumors. Different groups have reported the effectiveness of Dox also against GBM, both in preclinical models. Veringa et al. have demonstrated that Dox showed a high cytotoxic effect on primary GBM cell lines, more than conventional used TMZ and etoposide [4].

Our group have also recently investigated the efficacy of Dox treatment against GBM, reporting how Dox, in combination with other drugs, has a high cytotoxic effect against GBM cell lines and determined a decrease in volume by an orthotopic xenograft mice model of human GBM [5,6].

Cancer development is regulated by numerous and complex mechanisms. Hyperactivation of downstream phosphatidylinositol 3-kinase (PI3K)/AKT/ mammalian target of rapamycin (mTOR) pathway is a common occurrence of human glioma [7,8].

mTOR is a Ser/Thr protein kinase that plays a critical role in cellular responses to growth factors and stress, controlling mRNA translation, ribosome biogenesis, autophagy, and metabolism [9]. Rapa, also known as Sirolimus, is a clinically approved immunosuppressant that has shown promising antitumor activity. Rapa, a specific inhibitor of mTOR function, blocks serum-stimulated Ser-2448 phosphorylation by AKT and other downstream effectors, such as p70S6 kinase [10]. This drug has been shown to be effective against intracerebral glioma xenografts and has a cytostatic effect against gliomas [11]; several Rapa derivatives (rapalogues) have been synthesized, namely temsirolimus, everolimus, and ridaforolimus. Currently, more than 30 clinical trials involve Rapa or its derivatives in brain and Central Nervous System (CNS) cancer therapy.

Basing on these evidences, the purpose of our study was to investigate the in vitro and in vivo efficacy of the co-treatment Rapa *plus* Dox, in order to elucidate the potential of this combined treatment as available approach for GBM therapy.

## 2. Materials and Methods

### 2.1. MTT Assay

The effect of the treatments, on tumor cells growth, was measured using the MTT Cell Proliferation Assay (Cayman Chemical, Ann Arbor, MI, USA), following the manufacturer’s instructions.

Three human GBM cell lines, A172, U87MG, and T98G, were employed in this study (American Type Culture Collection (ATCC), Manassas, VA, USA). U87MG and T98G were grown in Eagle’s Minimum Essential Medium, while A172 was grown in Dulbecco’s Modified Eagle Medium. Each medium was supplemented with 10% fetal bovine serum and 1% penicillin-streptomycin. All cell lines were maintained in a humidified atmosphere of 5% CO_2_—95% air at 37 °C.

GBM cell lines were seeded at the following densities: A172 and T98G 3 × 10^3^ cells/well, U87MG, 5 × 10^3^ cells/well in 96-well plates. After 24 h, cells were treated with Rapa (10 nM, 100 nM, and 1 µM), Dox (0.8 µM), and their combinations for 24h, 48h, and 72 h.

Optical density (OD) of each well was measured on a MULTISKAN FC (Thermo Scientific, Waltham, MA, USA) microplate reader at a test wavelength of 550 nm. All experiments were performed three times in triplicate.

### 2.2. Intracellular Dox Accumulation

Dox cellular uptake was analyzed using a FACScan flow cytometer (Becton Dickinson, Mountain View, CA, USA), equipped with a 488 nm argon laser. The exponentially growing P-glycoprotein (P-gp) transfected MDCKII cells were treated with Rapa (10 nM, 100 nM, and 1 µM), Dox (0.8 µM), and their combinations for 2 h, according to the previously described method [6]. Dox fluorescence was measured at a flow rate of 8000 events/s. All experiments were performed two times in triplicate.

### 2.3. Immunoblotting

After washing cells with PBS 1X, monolayers of A172, U87MG and T98G cells were lysed in ice-cold RIPA buffer added with protease inhibitor cocktail (Sigma Aldrich, Saint Louis, MO, USA) and centrifuged at 4 °C for 10 min at 10,000× *g*. The protein content was determined by using a BCA protein assay kit (Thermo Scientific, Waltham, MA, USA).

SDS-polyacrylamide gel electrophoresis was performed using 6% and 4% acrylamide for the separating and stacking gel, respectively. Blots were blocked with blocker milk 5% solution and incubated overnight at 4 °C with anti-mTOR, anti-℗ser2448mTOR (phosphorylation on 2448 is a biomarker of mTOR activity), and anti-vinculin primary antibodies (Cell Signaling Technology, Danvers, MA, USA). Blots were further incubated with secondary antibodies conjugated with horseradish peroxidase (Bio Rad, Hercules, CA, USA) for 1 h at room temperature. Proteins revelation were performed on chemi-doc imaging system (Bio Rad, Hercules, CA, USA) and densitometric analysis were made using ImageJ software.

### 2.4. Animals

110 Foxn1 female nude mice were used in this study. Animals procedure of accommodation and care have been described in detail previously [6].

Briefly, mice were housed inside cages of polisulfone (4 mice/cage) with stainless steel cover-feed and sterilized and dust-free bedding cobs. Food and bedding were sterilized. Drinking water was supplied ad libitum and each mouse was daily offered a complete pellet diet (GLP 4RF21, Mucedola) throughout the study.

Biogem laboratories obtained the authorization for animal manipulation by the Italian Health Authority.

The Care and Husbandry of animals are in accordance with European Directives No. 2010/63 and with the Italian Regulatory system (D.L. vo No. 26, 4 March 2014). The official Biogem veterinarian approves all parts of this study concerning animal care.

Animals were inspected every day for mortality. Physical appearance, behavior, general and local clinical signs were also observed; all efforts were made to minimize the mice’s suffering and mice showing clinical signs of pain and distress were euthanized for humane reasons by CO_2_ inhalation. At the end of experiments, all mice were euthanized by CO_2_ inhalation.

### 2.5. Drugs and Reagents

Dox (Pfizer) was diluted with sterile Saline solution, at different concentrations, and administered intra vein (tail vein, IV) in a volume of 5 mL/kg. Rapa (AdooQ Bioscience, Irvine, CA, USA) 5 mg/kg was dissolved in a solution containing 2% DMSO, 30% PEG400, 5% Tween80, and 63% MilliQ water and intraperitoneally (IP) administered in a volume of 5 mg/kg. All chemicals and solvents were of the highest purity available from commercial sources and used without further purification.

### 2.6. Experimental Design

The procedure of tumor implantation has been described in detail previously [6].

Briefly, U87MG-luc2 (PerkinElmer Italia S.P.A., Monza, Italy), a human GBM cell line stably transfected with firefly luciferase gene (luc2), was used to establish the orthotopic glioma model. On the day of the tumor implantation, mice were micro-injected with 3 × 10^5^ U87MG-luc2 cells in the left lobe of brain with infusion of 1 μL/min (with Hamilton syringe). Following intracranial tumor injection, bioluminescence (BLI) acquisitions were performed, at day 0, 3, and 7, for baseline data. After tumor implantation (day +7), mice were sorted based on BLI Average Radiance and randomly allocated as follows:

- Group 1 (Ctr): naïve mice received physiological solution (5 mL/kg).
- Group 2 (Rapa 5 mg/kg): daily administrations from day 7 to 42.
- Group 3 (Dox 5 mg/kg): weekly administrations (days 7, 14, 21, 28, and 35).
- Group 4 (Dox 2.5 mg/kg): weekly administrations (days 7, 14, 21, 28, and 35).
- Group 5 (Dox 5 mg/kg+Rapa): weekly administrations of Dox and daily administrations of Rapa.
- Group 6 (Dox 2.5 mg/kg+Rapa): weekly administrations of Dox and daily administrations of Rapa.

Each experimental group contained 16 animals. Tumor growth and weight were carried out weekly and biweekly, respectively, for five weeks.

### 2.7. In Vivo Imaging

Tumor growth and response to the treatments were monitored by BLI acquisitions, using IVIS 200 Spectrum Imaging System (PerkinElmer, Waltham, MA, USA). BLI acquisitions were performed immediately after tumor cells injection (day 0 and +7) and then weekly until the end of experiment.

In detail, mice were IP administered with 10 mg/kg/10 mL of D-Luciferin (PerkinElmer (Waltham, MA, USA) Xenolight D-Luciferin Potassium Salt) and after 30 min were anesthetized by gas anesthesia (2% isoflurane).

All animals were placed into the IVIS 200 Imaging System to be imaged. BLI was expressed as average radiance in photons per s/cm^2^ per steradian.

### 2.8. Body Weight

Body weight loss percent (% BWL) was calculated as follows: 100 − (mean BW day-x/mean BW day-1 × 100). Measurements were carried out biweekly and, as humane endpoint, animals showing a BWL ≥ 15% were euthanized by CO_2_ inhalation.

### 2.9. Histological Evaluation

At day +42, brains of 48 mice (8 animals/group) were collected, weighed, and preserved in 10% neutral buffered formalin solution (Sigma Aldrich, Saint Louis, MI, USA), in order to accomplish morphological studies. Based on BLI signals, the most representative five brains of each group were selected and submitted to the internal standard trimming, wax embedding, and cutting procedures. Serial slides were stained with haematoxylin-eosin or Ki67 (Dako, Santa Clara, CA, USA) immunostaining, in order to calculate the proliferation index within the tumor mass.

### 2.10. Proliferation Index—Ki67 Quantitative Analysis

Among the 30 embedded brains, the most representative specimens, at least three for each group, were considered for the proliferation index analysis.

In order to intercept tumor mass, samples were explored at different cutting levels; sections were collected and examined at regular intervals of 50 μm.

Ki67 immunodetection was performed on tumor-bearing specimens, identified on haematoxilyn-eosin stained slides; single or multiple sections were subjected to Ki67 immunohistochemistry, in order to reach the required number of nuclei.

Ki67 immunohistochemistry was performed by primary anti-human Ki67 mouse monoclonal antibody (Dako 1:100), incubated for 1 h at room temperature in antibody diluent (Leica Biosystems, Wetzlar, Germany, EU). The immunodetection technique was conducted with NovoLink Polymer Detection Systems kit (Leica Biosystems, Wetzlar, Germany, EU); Rodent Block M (Biocare Medical, Pacheco, CA, USA) was used for blocking endogenous mouse IgG and non-specific binding sites. Negative control (primary antibody omitted) was also performed.

Ki67 quantitative analysis was performed in representative fields of tumor mass, acquired at final magnification of 400×; 1000 tumor nuclei/case were counted. Proliferation index was expressed as the percentage of Ki67-positive tumor nuclei over the total number of neoplastic nuclei. For each case, the mean value was assigned as individual data; cell positivity was evaluated with TMarker software v2.146 version, a free tool for histopathological cell counting and staining estimation.

### 2.11. Statistical Analysis

Data were expressed as mean ± SD. Statistical analyses were performed using T-test between two groups while comparison between multiple groups were performed using 1- or 2-way ANOVA followed by post hoc corrections, as appropriate.

For the evaluation of in vivo results, outliers were removed through ROUT test (95% confidence interval) and the statistical analysis was performed using the unpaired Mann–Whitney U-test.

For histological analysis, outliers were removed through ROUT test (95% confidence interval), and statistical analyses were performed with the unpaired Mann–Whitney U-test or ANOVA followed by Tukey correction, as appropriate. All analyses were done using GraphPad Prism 5 and *p* < 0.05 was considered statistically significant.

## 3. Results

### 3.1. Cytotoxic Effect of Combined Treatments Rapa Plus Dox in GBM Cell Lines

**Figure 1:**
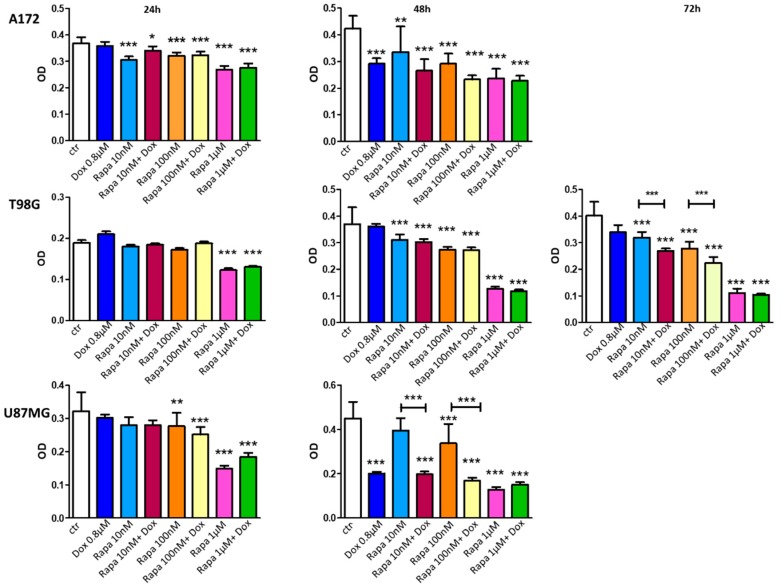
Effect of Dox, Rapa and their combinations on human glioblastoma (GBM) cell growth. U87MG, A172, and T98G cells were treated with various concentrations of drugs for 24 h and 48 h. In T98G Dox resistant cells, the effects of Dox, Rapa, and their combinations have been also evaluated after 72 h of treatment. Statistical analysis was performed with 1-way ANOVA followed by Bonferroni post hoc correction (* *p* < 0.05; ** *p* < 0.01; *** *p* < 0.001; **** *p* < 0.0001 vs. control group). Data were expressed as mean ± SD. This experiment was performed three times in triplicate.

To assess the cytotoxic effects of the treatments, three GBM cell lines (A172, U87MG, and T98G) were exposed to Rapa (10 nM, 100 nM, and 1 µM), Dox (0.8 µM), and their combinations, at different time points (Figure 1).

Results obtained in A172 cells, after 24 h of treatments: Dox has no effect compared to control. All treatment including Rapa showed significant statistical differences vs. control. In conclusion, these data reported that the cytotoxic effect was related only to Rapa.

Results obtained in A172 cells, after 48 h of treatments: all treatments have significant effects vs. control. Comparing Rapa and Rapa *plus* Dox treatments, no significant differences were reported but we can observe a clear incremented cytotoxic effect in co-treated groups compared to Rapa and Dox single treatments.

Results obtained in T98G cells, after 24 h of treatments: all treatments have no effect vs. control, excluding Rapa 1 µM and Rapa 1 µM *plus* Dox (0.1231 ± 0.01356; 0.1304 ± 0.007091 vs. 0.1889 ± 0.01972, *** *p* < 0.001). No statistically significant differences were observed comparing between these two groups, indicating that the cytotoxic effect of the treatments was related only to Rapa.

Results obtained in T98G cells, after 48 h of treatments: as previously reported [5], these cells remained resistant to Dox treatment while all other treatments showed significant effects vs. control. No significant differences were observed comparing between Rapa and Rapa *plus* Dox groups, at all concentrations, confirming that the cytotoxic effect was related only to Rapa.

Results obtained in T98G cells, after 72 h of treatments: once again, cells remained resistant to Dox treatment while all other treatments showed significant effects vs. control. Moreover, statistically significant results were obtained comparing between Rapa 10 nM and Rapa 100 nM vs. Rapa *plus* Dox treatments (0.2691 ± 0.009347 and 0.2244 ± 0.02207 vs. 0.3399 ± 0.02708 respectively; **** *p* < 0.0001). OD value of Rapa 1 µM *plus* Dox treatment was lower than Rapa 1 µM alone but the difference was not statistically significant (Figure 1). Taken together, these results indicated that prolonged exposure to Rapa probably sensitizes T98G to Dox-mediated cytotoxic effect.

Results obtained in U87MG cells, after 24 h of treatments: Dox has no effect vs. control. Rapa alone and in combination with Dox showed significant statistical differences vs. control only at higher concentrations but no significant statistical differences were reported comparing Rapa alone vs. Rapa *plus* Dox, at all concentrations. As observed for A172, at 24 h of treatment, the cytotoxic effect was related only to Rapa.

Results obtained in U87MG cells, after 48 h of treatments: Dox alone has a marked significant effect vs. control (0.2009 ± 0.007373 vs. 0.4487 ± 0.07439, *** *p* < 0.001). Rapa 10 nM remained ineffective vs. control while Rapa 100 nM and 1 µM effects resulted in slight improvement compared to 24 h. Significant statistical differences can be observed in all co-treated groups vs. control. Moreover, significant statistical differences were observed comparing Rapa 10 and 100 nM vs. respective Rapa *plus* Dox groups (*** *p* < 0.001) but, no difference were revealed comparing Dox vs. Rapa *plus* Dox combinations. Opposite to 24 h, 48 h data reported that Dox was majorly responsible for the cytotoxic effect of the treatments.

### 3.2. Effect of Rapa Treatments on Dox Uptake

Drug passage through blood brain barrier (BBB) is regulated by the presence of efflux transporters as P-gp. To determinate if Rapa treatments were able to influence Dox passage across cell membranes, we have investigated the cellular uptake of the anthracycline by using MDCKII cell line, a largely approved in vitro model of BBB.

**Figure 2:**
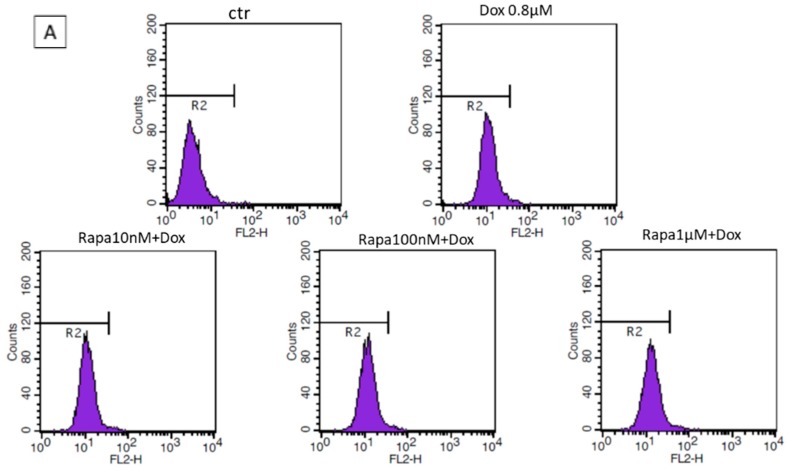

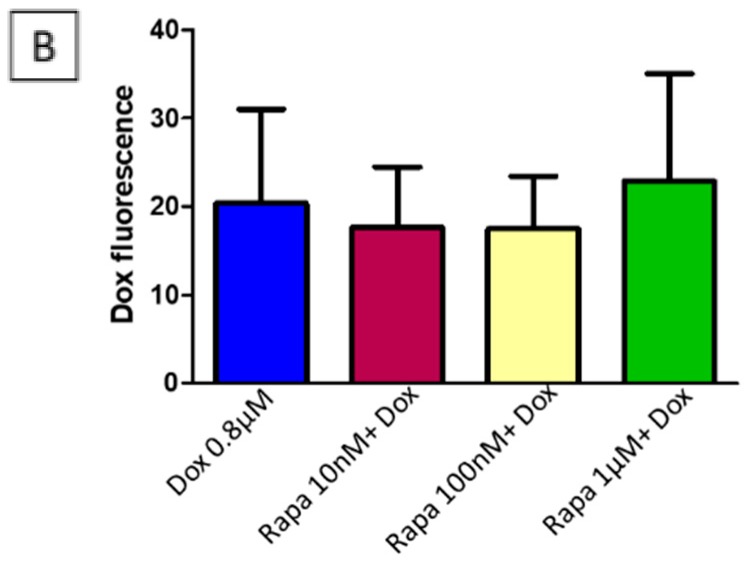
Effect of Rapa on Dox uptake in MDCKII P-gp transfected cells. (**A**) Cellular uptake of Dox by flow cytometry. Each peak represents the mean fluorescence intensity of Dox (0.8 μM) in control and treated samples; (**B**) No significant difference can be observed comparing Dox fluorescence signals between cells treated with Dox (0.8 µM) vs. Rapa *plus* Dox. Statistical analysis was performed by 1-way ANOVA followed by Bonferroni post hoc correction. Data were expressed as mean ± SD. This experiment was performed two times in triplicate.

Dox is an autoflorescent molecule and its intracellular accumulation is highly influenced by the presence of the P-gp protein [6], for this reason, we utilized MDCKII- P-gp transfected cells for our experiments. As reported in Figure 2A,B, no statistical differences were observed comparing Dox vs. Dox *plus* Rapa treated samples.

This data showed that Rapa, at all tested concentrations, not influence P-pg efflux activity and subsequently Dox uptake in MDCKII P-gp transfected cells. However, an incremented Dox cell accumulation can be appreciated in the presence of the higher Rapa concentration.

### 3.3. Effect of Dox plus Rapa Treatments on mTOR Expression

**Figure 3:**
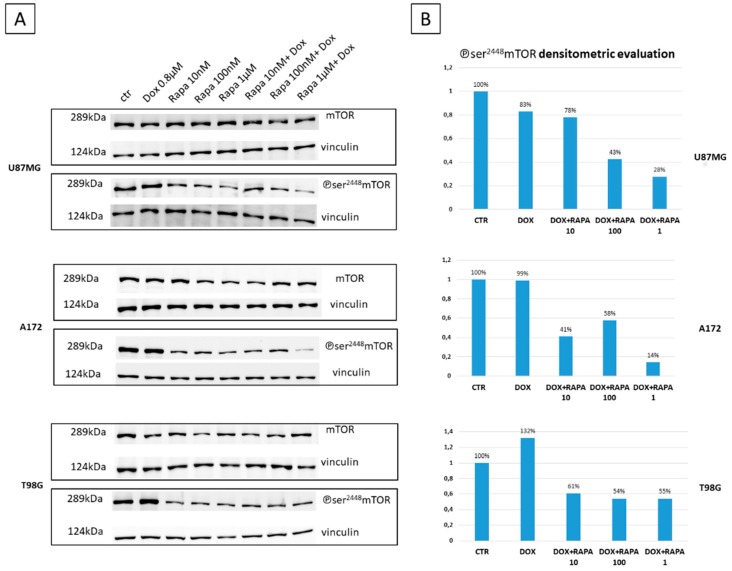
Effect of Rapa on mTOR S2448 phosphorylation (**A**) Immunoblot analysis of mTOR and ℗ser^2448^mTOR signaling in U87MG, A172, and T98G glioma cells, treated with Dox, various concentrations of Rapa and their combinations for 8 h. Vinculin was used as a protein loading control; (**B**) Densitometric analysis of ℗ser^2448^mTOR signaling in U87MG, A172, and T98G glioma cells. All samples were normalized vs. vinculin and ℗ser^2448^mTOR levels were referred to CTR (100%).

Immunoblotting results on U87 MG and A172 (a Dox sensible GBM cell lines) demonstrated that Dox has no effect on mTOR activity (phosphorylation of Ser2448) (Figure 3A). Meanwhile, as expected, Rapa treatments were able to reduce the phosphorylation of Ser2448, in both cell lines (Figure 3A). The most interesting results were obtained analyzing the effect of the combined treatments. Densitometric evaluation showed that Rapa 10 and Rapa 100 *plus* Dox reduced the activity of mTOR (U87MG:78% and 43% phosphorylation of Ser2448 vs. control; A172:41% and 58% phosphorylation of Ser2448 vs. control) while, Rapa 1 *plus* Dox displayed a substantially inhibition of the kinase activity (U87MG: 28% phosphorylation of Ser2448 vs. control; A172: 14% phosphorylation of Ser2448 vs. control) (Figure 3B).

Densitometric evaluation of mTOR activation in T98G (a Dox resistant GBM cells) revealed that treatment with Dox alone increment phosphorylation of Ser2448 (132% vs. control), while its combination with Rapa induced a ~50% reduction of mTOR activation (Figure 3B).

### 3.4. Effect of Dox Plus Rapa Treatments on Glioblastoma Growth in Mouse Xenograft Model

**Figure 4:**
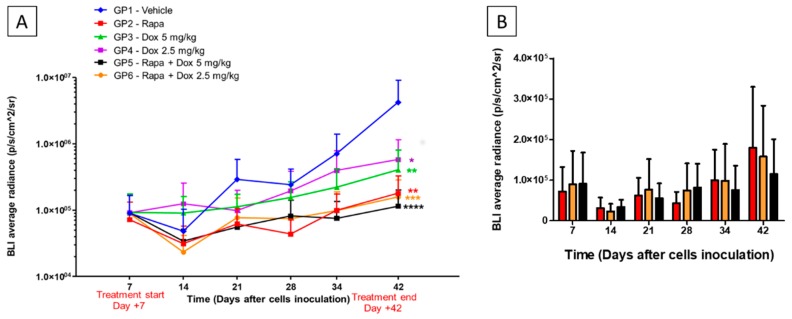

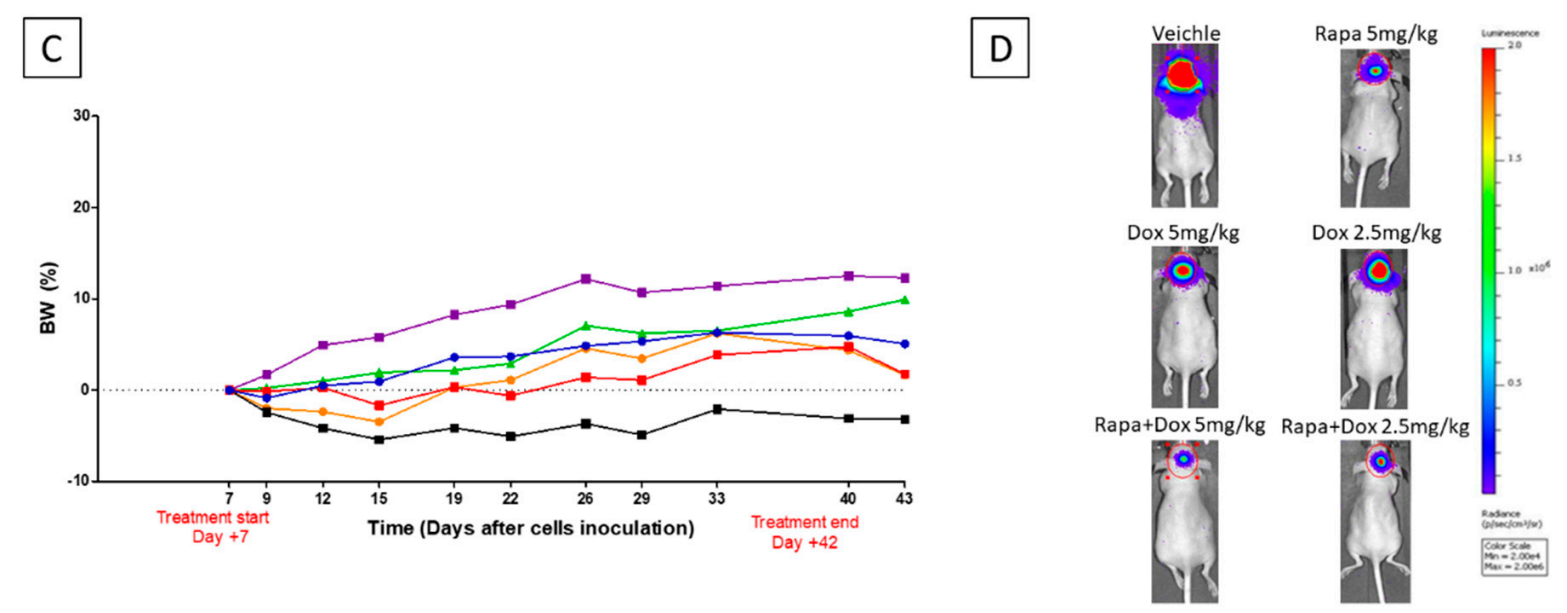
Bioluminescence signals throughout the experimental period. (**A**) Comparison of BLI acquisitions between all experimental groups. Outliers were removed by using the ROUT test (Q = 1%). Statistical analysis was performed by Mann–Whitney U-test at day +42 (end of treatment) (* *p* < 0.05; ** *p* < 0.01; *** *p* < 0.001; **** *p* < 0.0001 vs. Vehicle-treated group); (**B**) BLI trends in GP2, GP5, and GP6 throughout the experimental time; (**C**) Percent of body weight variation throughout the experimental period; (**D**) Representative images of BLI acquisition of each groups at day +42 (end of treatment).

One week after tumor implantation, 96 animals were divided into six groups and treated following the experimental schedule as described in Materials and Methods section. Treatments time-course and BLI evaluation of tumor growth are reported in Figure 4A.

Statistically significant results were obtained comparing treated groups vs. control group, at day +42.

At day +42 the BLI Average Radiance of control group was 4.25E+06 vs. 2.48E+05 of Rapa group (** *p* < 0.01), 4.07E+05 of Dox 5 mg/kg group (** *p* < 0.01), 1.10E+06 of Dox 2.5 mg/kg group (* *p* < 0.05), 1.36E+05 of Rapa *plus* Dox 5 mg/kg (**** *p* < 0.0001) and 5.13E+05 of Rapa *plus* Dox 2.5 mg/kg group (*** *p* < 0.001). At day +42, two animals from the control group died before BLI acquisition.

The tumor volume inhibition, determined by the various treatments, indicated that Rapa and Dox, alone or in combination, showed a significant effect against GBM growth (Figure 4A).

All treatments determined a tumor growth inhibition from 86% to 97% but the most significant result has been given by Rapa *plus* Dox 5 mg/kg combination (−97.29%). The in vivo results also revealed that the combination with Dox slight sensitized GBM to the cytotoxic effect of Rapa (Rapa −95.76%, Rapa *plus* Dox 2.5 mg/kg −96.28%, Rapa *plus* Dox 5 mg/kg −97.29%). Moreover, in accordance with in vitro results, Figure 4B shows BLI trends of GP2, GP5 and GP6 during the experimental time. In this figure, is possible to appreciate the total inversion of BLI value that occurs between these groups, underling the concept that even if Rapa alone appear to be effective against GBM its effect is maximal in the first stage, while prolonging treatments, the effectiveness of Rapa decreased and combined treatments showed a higher BLI reduction.

Side effects of the treatments, evaluated as BW% variations of treated groups compared to control group, are reported in Figure 4C.

At day +43, we reported a BW% variation of 1.75% in Rapa group, 9.91% in Dox 5 mg/kg group, 12.32% in Dox 2.5 mg/kg group, −3.19% in Rapa *plus* Dox 5 mg/kg group and 1.64% in Rapa *plus* Dox 2.5 mg/kg group.

This data indicated that, even if Rapa *plus* Dox 5 mg/kg treatment shows the highest effect on tumor growth inhibition (Figure 4D), this combination was the only one that caused weight loss (with a negative BW% variation), resulting in greater toxicity.

### 3.5. Proliferation Index—Ki67 Quantitative Analysis

At the end of the treatments (day +42) brains of five animals of each group were collected to undergo histological evaluation.

Samples were explored at different cutting levels and serial slides were stained with haematoxylin-eosin or Ki67 immunostaining, in order to calculate the proliferation index within the tumor mass.

**Figure 5:**
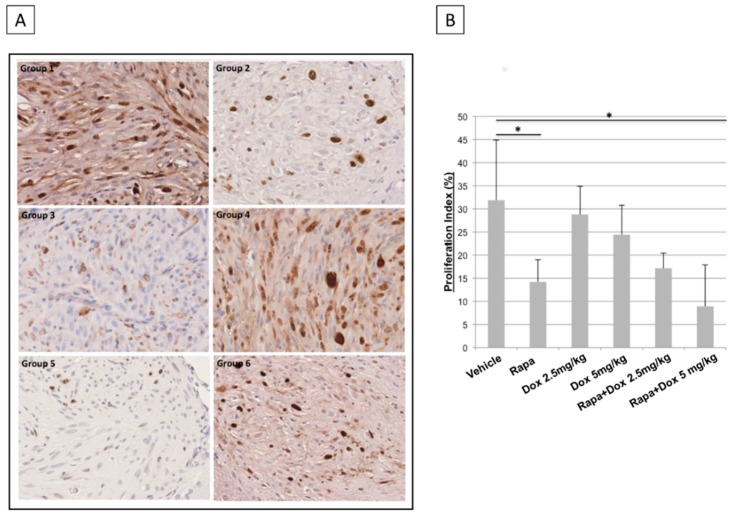
Immunohistochemical evaluation of therapies on glioma proliferation (**A**) Representative images of Ki67-immunoreactivity showed by tumor samples from each groups. Formalin-fixed, Wax-embedded, anti-Ki67 IHC stained slides—Final Magnification 400×; (**B**) Proliferation index comparison between vehicle group vs. treated groups; statistical analysis was performed through the unpaired Mann–Whitney U-test and ANOVA with Tukey correction (* *p* < 0.05).

As reported in Figure 5A,B, Rapa monotherapy induced a meaningful and statistically significant decrease in the proliferation index compared to control samples, while Dox (5 and 2.5 mg/kg) induced a slight and not significant reduction of the value in monotherapy regimen.

Combined treatments showed better results than monotherapies. In particular, Rapa *plus* Dox 2.5 mg/kg samples present a proliferation index comparable to Rapa treatment while, Rapa *plus* Dox 5 mg/kg result the best treatment with the lowest value of proliferation and a statistically significant difference compared to control (8.92 ± 3.3 vs. 31.87 ± 12.98; * *p* < 0.05) (Figure 5B).

## 4. Discussion

The role of chemotherapy for the treatment of GBM patients remains an ongoing challenge for the scientific community.

Numerous drugs have shown great toxicity in GBM in vitro models, but clinical trials, using consolidated and new agents, have showed no significant improvement in terms of overall survival and progression-free survival [12]. These disappointing results can, at least in part, be explained by (i) the inability to deliver more effective anticancer agents, as Dox, into the CNS across the BBB, avoiding various resistance mechanisms, including the active efflux of anticancer drugs mediated by ATP-binding cassette (ABC) transporters as P-gp [13]; (ii) the acquisition of adaptive and resistance mechanisms of GBM cells in the presence of prolonged treatment with metabolic activity modulators, such as Rapa [14].

It is becoming clear that combination or sequential treatment modalities, that target multiple pathways, can lead to better control of aberrant cell proliferation.

We have investigated the potential of a new pharmacological approach, combining Dox, an anthracycline more effective than TMZ against GBM [4,15] and Rapa, a potent inhibitor of mTOR that is largely involved in GBM proliferation and progression [16].

In accordance with literature data [17], our in vitro results demonstrated the cytotoxic effect of Rapa on GBM cell lines with different degrees of sensibility. At 24 h of treatment, the cytotoxic effect can be attributed exclusively to Rapa; significance was obtained only in treatments including Rapa vs. controls. In the same cell lines, excluding T98G (that we have previously reported to be resistant to Dox [5]), Dox effectiveness was appreciable only 48 h after treatments. This data indicated that Rapa blocks cells proliferation in a short period while Dox cytotoxic mechanism requires more time to induce GBM cell death. An interesting result was obtained in T98G cells, prolonging treatments at 72 h.

In this case, T98G remained resistant to Dox but showed a high cytotoxic effect when co-treated with Dox and Rapa; the most interesting result was the significance achieved comparing Rapa vs. Rapa *plus* Dox groups, suggesting that Rapa probably sensitizes T98G cells to Dox.

Moreover, we have investigated the mechanism underlying the increased cytotoxicity of the combined treatment Rapa *plus* Dox compared with only Dox focusing our attention on two goals: the effect of treatments on mTOR modulation and effect of treatments on Dox uptake.

mTOR is a critical mediator of numerous cellular signals in oncogenesis [8] and consists of two distinct components: mTORC1 and mTORC2. Rapa-sensitive mTORC1 directly targets ribosomal protein S6 kinase (p70S6K) and eukaryotic translation initiation factor 4E-binding protein 1 (4E-BP1) to promote cap-dependent protein translation [18,19]. At molecular level, Ser2448 is the most studied phosphorylation sites on mTORC1. Recent studies have demonstrated that S2448 phosphorylation is a biomarker of mTORC1 activity [20].

We reported a clear modulation of the combined treatments on S2448 phosphorylation, vs. control and Dox treatment, in GBM cell lines (Figure 3B). The effect was more appreciable in U87MG and A172, Dox sensible cells, with a maximum phosphorylation inhibition associated with Dox *plus* Rapa 1 µM co-treatment (72% and 86% vs. control).

T98G, a Dox resistant cell line, showed a minor modulation with a ~50% S2448 phosphorylation inhibition. This 50% mTOR conserved activity in association with Dox resistance can be identified as contributing causes of T98G multidrug resistance (MDR) phenotype. These interesting results, in association with cytotoxic data, clearly show that MDR is a complex phenotype, mediated from different factors and pathways. Combinational or sequential treatment modalities that investigate multiple targets can lead to better control of aberrant cell proliferation.

Interestingly, Arcella et al. also found that, despite producing mTOR inhibition, Rapa administration did not produce a compensatory increase in Akt levels. In detail, they found that Rapa robustly decreased phosphorylated (p)Akt levels while non-phosphorylated Akt levels were steady, producing a decrease in the ratio between pAkt and Akt. These data were consistent along patients and U87MG cell line [14].

Literature data about the interaction between Rapa and P-gp, the major responsible of drug efflux at BBB level, are currently conflicting.

Different studies reported that Rapa and its analogue, Tacrolimus, tested at 2.5 µM concentration, were able to modulate P-gp and BCRP activity [21,22].

On the other hand, in vitro data on fibrosarcoma, leukaemia, and immune cells reported that Rapa did not modulate P-gp, MRP-1, or BCRP at its clinically achievable concentration of 0.25 µM. The same result was also obtained using a Rapa analogue, Tacrolimus, at its clinically achievable concentration of 0.08 µM.

In accordance with these outcomes, we also report comparable data evaluating the effect of Rapa on Dox uptake by using an in vitro model of BBB. Dox is a P-gp substrate and, taking advantage of its autofluorescence, we have quantified Dox intracellular accumulation in presence of various doses of Rapa in MDCKII P-gp transfected cells. As reported in Figure 3B, no differences in Dox signal were observed comparing all treated samples vs. control and neither vs. Dox only. This result indirectly demonstrated that Rapa (at tested concentrations) has no effect on Dox P-gp-mediate transport across BBB (Figure 2B).

These results indicated that the affinity and modulatory effect of Rapa, on the ABC transporter, was strongly related to the used dosage of the mTOR inhibitor.

Considering the interesting in vivo results obtained by Arcella et al., we have tested the efficacy of the co-treatment Rapa *plus* Dox in a xenograft mice model of human GBM. They reported a strong efficacy of Rapa (5 mg/kg) on cell proliferation, tumor volume inhibition, and survival. Therefore, we tested the same dose for our study as well. [14]. Dox doses, for in vivo experiments, have been replaced from our previous experiments [6].

At the end of the experiment, day +42, our results indicated that all treatments had noteworthy effects on BLI and tumor volume inhibitions, with different degrees of significance (Figure 4A). The most effective approaches resulted, for both treatments, Rapa *plus* Dox 5 mg/kg and Rapa *plus* Dox 2.5 mg/kg. These results were also supported by immunohistological analysis. Nuclear Ki-67 immunopositivity is higher in rapidly growing malignant tumors than in their slowly growing benign counterparts [23,24,25].

Five brains for each group undergoes to histological evaluation. The difference in the number of the examined samples is due to the difficult interception of the tumor mass, especially in the co-treated groups. Our samples were stained with haematoxylin-eosin or Ki67 immunostaining, in order to calculate the proliferation index within the tumor mass (Figure 5A). Rapa *plus* Dox 2.5 mg/kg samples presented a proliferation index comparable to Rapa treatment while, Rapa *plus* Dox 5 mg/kg result in the best treatment with the lowest value of proliferation and a statistically significant difference compared to control.

Rapa *plus* Dox 5 mg/kg resulted in the best treatment under different points of view, but it was associated with a greater toxicity, evaluated as BW% variation. Interestingly, even if Rapa had a very slight effect on Dox uptake (Figure 2), the combination Rapa *plus* Dox 2.5 mg/kg showed a tumor inhibition like Rapa *plus* Dox 5 mg/kg combination. It is likely that in the presence of Rapa adjuvant effects, the Dox result is able to express its cytotoxic potential at lower concentrations, with reduced side effects.

In conclusion, our results demonstrate that Rapa *plus* Dox treatment had the advantage (i) to reduce tumor cells proliferation by reducing the mTOR activation; (ii) to improve the cytotoxic effect of Dox; and (iii) to reduce the risk for a clinically relevant negative impact of protective effects of mTOR modulation, combining the antiproliferative/cytotoxic effect of Rapa with the cytotoxic effect of Dox (iiii) to make possible a reduction of the anthracycline dosage and consequently, Dox-mediated side effects of the treatments.

Further studies are necessary to better understand the potentiality of the combined treatment Rapa *plus* Dox, but this safe and non-invasive approach could be a successful strategy in the treatment of aggressive tumors as GBM.
